# Accelerated midlife endocrine and bioenergetic brain aging in APOE4 females

**DOI:** 10.3389/fnagi.2025.1632877

**Published:** 2025-08-18

**Authors:** Tian Wang, Zisu Mao, Yuan Shang, Simona Merlini, Francesca Vitali, Jean-Paul Wiegand, Roberta Diaz Brinton

**Affiliations:** ^1^Center for Innovation in Brain Science, University of Arizona, Tucson, AZ, United States; ^2^Department of Neurology, University of Arizona, Tucson, AZ, United States; ^3^Department of Biomedical Engineering, University of Arizona, Tucson, AZ, United States; ^4^Department of Pharmacology, College of Medicine Tucson, University of Arizona, Tucson, AZ, United States

**Keywords:** Menopause, APOE4, women, Alzheimer′s, mitochondrial function, neuroinflammation

## Abstract

Female sex, age, and APOE4 genotype are the greatest risk factors for Alzheimer′s disease. Using a translational perimenopause mouse model based on human Stages of Reproductive Aging Works (STRAW) criteria, we investigated the impact of APOE genotype on female midlife endocrine aging, peripheral metabolic indicators, brain bioenergetic pathways, mitochondrial function, neuroimmune activation, and myelination. Compared to APOE3 females, APOE4 females exhibited accelerated endocrine aging that was coincident with failure to mount adaptive bioenergetic reprogramming and significant decline in mitochondrial function that were coupled with increased immune activation and demyelination in brain. In women, APOE4 was associated with early menopause. Further, APOE4 women experiencing early menopause exhibited the highest risk of Alzheimer′s. These results provide plausible mechanistic pathways underlying the earlier emergence and greater risk of Alzheimer′s in APOE4 postmenopausal females. Collectively, these findings support midlife as a critical window for intervention to prevent or delay the onset of the prodromal stage of Alzheimer′s disease in APOE4 carriers.

## 1 Introduction

Worldwide, there are currently over 850 million women 40–60 years of age (United States Census Bureau, 2014), which corresponds to the midlife endocrine transition of the perimenopause to menopause (Brinton et al., 2015). Midlife endocrine aging is experienced by women across the globe (Brinton et al., 2015). As a national example of incidence and prevalence, each year ∼1.5 million American women enter into the perimenopause and in 2020 there were 45 million US women over the age of 55 (United States Census Bureau, 2017).

The greatest risk factors for Alzheimer′s disease (AD) are age, APOE4 allele, and female sex (Altmann et al., 2014, Farrer et al., 1995, Hebert et al., 2013, Sala Frigerio et al., 2019, Payami et al., 1996, Barnes et al., 2005, Nebel et al., 2018). Approximately two-thirds of the Alzheimer′s population are women and women bear the greatest burden of the disease (Brinton, 2008; Brookmeyer et al., 2007, Morrison et al., 2006, Brookmeyer et al., 1998; Alzheimer’s Association, 2019). In the brain, the perimenopause to menopause transition impacts neural circuits beyond those associated with reproduction (Brinton et al., 2015). In the aggregate, the menopausal transition fulfills criteria for a critical period of aging in women (Brinton et al., 2015). Multiple conditions that can emerge during the menopausal transition are associated with increased risk of AD (Brinton et al., 2015) and/or development or exacerbation of autoimmune diseases (Desai and Brinton, 2019). In both pre-clinical and human studies, disrupted estrogen regulation during perimenopause results in decreased brain glucose metabolism and mitochondrial function, increased neuroinflammation, and increased AD-related pathology (Yin et al., 2015, Wang et al., 2020b; Brinton et al., 2015, Klosinski et al., 2015, Bacon et al., 2019, Mosconi et al., 2017a,Mosconi et al., 2017b). Additionally, estrogen loss induced by ovariectomy in animal models has been shown to impair brain glucose metabolism, induce mitochondrial dysfunction and oxidative stress, increase glial activation, and exacerbate amyloid-β accumulation, synaptic loss, and cognitive deficits (Itoh et al., 2023, Luo et al., 2022, Yao et al., 2012). Remarkably, but perhaps not surprisingly, the 15–20 yr interval between menopause and AD diagnosis coincides with the prodromal phase of AD, during which pathological changes in brain occur before clinical diagnosis (Amieva et al., 2008, Vermunt et al., 2019, Villemagne et al., 2013, Mosconi et al., 2017a,Mosconi et al., 2018, Wang et al., 2020a).

APOE4 increases the risk of late-onset AD up to 15-fold in homozygotes (Yamazaki et al., 2019) and is associated with an earlier age of disease onset (Sando et al., 2008). Over 60% of persons with AD are APOE4 carriers (Riedel et al., 2016). In human brain, APOE4 carriers exhibit accelerated generation of amyloid-β, increased p-tau accumulation, accompanied by an earlier onset of reduced brain glucose metabolism and impaired mitochondrial function (Reiman et al., 2001, Reiman et al., 2004, Mosconi et al., 2004, Mosconi et al., 2005, Valla et al., 2010, Wolf et al., 2013, Yamazaki et al., 2019, Drzezga et al., 2005, Baek et al., 2020). Mechanistically, APOE4 contributes to AD pathogenesis evidenced by enhanced amyloid-β aggregation (Yamazaki et al., 2019, Verghese et al., 2013, Kanekiyo et al., 2014), exacerbated tau pathology (Shi et al., 2017), impaired glucose metabolism and lipid homeostasis (Wu et al., 2018, Qi et al., 2021, Zhao et al., 2017), altered microglial response (Kanekiyo et al., 2014, Fernandez et al., 2019), reduced synaptic integrity and plasticity (Dumanis et al., 2009, Ji et al., 2003, Klein et al., 2010), and disrupted cerebrovascular integrity and function (Koizumi et al., 2018, Halliday et al., 2016).

Multiple studies indicate a strong interaction between APOE4, female sex, and AD risk (Mishra and Brinton, 2018, Mishra et al., 2022, Shang et al., 2020, Wang and Brinton, 2016, Riedel et al., 2016). A meta-analysis of 27 independent studies in the Global Alzheimer′s Association Interactive Network recently confirmed that APOE4 + women exhibit an accelerated trajectory in AD odds ratio between 65 and 75 years of age (Neu et al., 2017). Further, APOE4 significantly exacerbated amyloid-β deposition (Mosconi et al., 2017b) and accelerated rates of cognitive decline in women (Holland et al., 2013). The APOE4-sex interaction is also evident in APOE mouse models, with more pronounced APOE4-induced neurodegeneration and cognitive deficits in female mice (Raber et al., 1998, Ungar et al., 2014, Rijpma et al., 2013).

To investigate the impact of APOE genotype on midlife endocrine aging, we utilized humanized targeted replacement of APOE3 and APOE4 mice to develop a translational mouse model of midlife female endocrine aging (the perimenopause animal model, PAM) based on the validated STRAW criteria developed for women (Bacon et al., 2019, Brinton et al., 2015, Mishra and Brinton, 2018, Wang et al., 2020b,Yin et al., 2015, Mishra et al., 2020). We characterized the endocrine status, plasma hormone and biometric profiles, brain bioenergetic function, myelination, and neuroinflammation. To delineate potential underlying mechanisms mediating the effects of menopause and APOE genotype on brain aging, we analyzed the transcriptomic and proteomic profiles of key metabolic and immune regulators in brain. Outcomes of these analyses revealed that APOE4 female mice exhibited accelerated endocrine aging coupled with an amplified bioenergetic crisis, myelin degeneration, and immune activation. We further investigated the translational significance of our findings using UK Biobank data. APOE4 was associated with early menopause in women. Further, APOE4 women with early menopause exhibited the highest risk of Alzheimer′s. Collectively, these findings provide mechanistic insights relevant to the increased risk of AD in APOE4 women.

## 2 Materials and methods

### 2.1 Animals

All animal studies were performed following National Institutes of Health guidelines on the use of laboratory animals and all protocols were approved by the University of Arizona Institutional Animal Care and Use Committee. Humanized APOE4 targeted replacement (APOE4) homozygous mice were obtained from Jackson Laboratory (#027894). Humanized APOE3 targeted replacement heterozygous mice were obtained from Jackson Laboratory (#029018) and bred to get homozygous APOE3 mice. Mice were housed on 14 h light/10 h dark cycles and provided *ad libitum* access to food and water.

### 2.2 Perimenopausal animal model

The estrous cycle status of 6, 9, and 14–15 months old APOE3 and APOE4 females were monitored by daily vaginal cytology. Vaginal smears were obtained between 0800 and 1100 h. Four stage of estrous cycle: Estrus (E), Metestrus (M), Diestrus (D), and Proestrus (P) were morphologically characterized based on the proportion of different cell types presented in the smears as previously described (Wang et al., 2020b,Yin et al., 2015). Female middle-aged mice were then stratified into 3 different endocrine aging groups with defined stages as per STRAW criteria (Harlow et al., 2012): regular cyclers (4–5 day cycles), irregular cyclers (6–9 day cycles), and acyclic (no cycling within 9 days). Typically, mice transit from regular cyclers to irregular cyclers at around 9 months (Finch, 2014). Thus, to capture this endocrinological transition, separate age-matched cohorts of APOE3 and APOE4 female mice were monitored, characterized, and collected at 3 time points: 6 months (young, 6M-Reg), 9 months (early perimenopausal transition, 9M-Reg and 9M-Irreg) and 15 months (late perimenopausal transition, 15M-Irreg and 15M-Acyc) for this study. Mice that did not meet the endocrine status criteria were excluded from the study.

### 2.3 Body composition measurement

Body composition was measured using EchoMRI™-700 (EchoMRI™) whole body composition analyzer following manufacturer’s instructions.

### 2.4 Blood and brain tissue collection

Mice were fasted overnight prior to euthanasia. Blood was collected through cardiac puncture into 1.5 mL EDTA-coated Eppendorf tubes. Blood samples were gently mixed and stored on ice for 30 mins, then centrifuged at 1,200 g for 15 mins at 4°C. The supernatant (plasma) was transferred into new Eppendorf tubes and stored in −80°C for subsequent assays. The brain was removed and dissected on ice. Brain tissue was snap frozen on dry ice and stored in −80°C for subsequent assays.

### 2.5 Peripheral metabolic markers

Fasting glucose level was measured by glucose meter (Abbott, 70804 and Abbott, 70819-70) through tail vein bleeding. Fasting plasma triglyceride levels (Cayman Chemical, 10010303) and ketone body levels (Cayman Chemical, 700190) were measured by colorimetric assays following manufacturer’s instructions.

### 2.6 Plasma estradiol and progesterone measurement

Plasma estradiol and progesterone levels were measured using Calbiotech Mouse/Rat Estradiol ELISA kit (ES180S-100) and Progesterone ELISA kit (PG362S) following manufacturer’s instructions.

### 2.7 Inflammatory biomarkers measurement

Inflammatory biomarker levels were determined by V-PLEX plus proinflammatory panel 1 (mouse) kit (Meso Scale Discovery, K15048G) according to manufacturer’s instructions.

### 2.8 Mitochondrial DNA copy number measurement

Total DNA was isolated from cortex tissues with QIAamp DNA mini kit (Qiagen, Valencia, CA) and analyzed by quantitative PCR. Relative mtDNA/nDNA ratio was calculated as the relative fold change of mt-ND1 (mtDNA) content to HK2 (nDNA) content as previously described (Wang et al., 2019). Primers were as follows: mt-ND1 forward: 5′-CTAGCAGAAACAAACCGGGC-3′; mt-ND1 reverse: 5′-CCGGCTGCGTATTCTACGTT-3′; nHK2 forward: 5′-GCCAGCCTCTCCTGATTTTAGTGT-3′; and nHK2 reverse: 5′- GGGAACACAAAAGACCTCTTCTGG-3′.

### 2.9 Western blot analysis

Protein concentrations were determined by using the BCA protein assay kit (Pierce, Rockford, IL). Equal amounts of proteins (20 μg/well) were loaded in each well of a 4–15% SDS-PAGE gel, electrophoresed with a Tris/glycine running buffer, and transferred to a 0.2 μm pore size polyvinylidene difluoride (PVDF) membrane and immunoblotted with the following primary antibodies: mouse total OXPHOS rodent WB antibody cocktail (Abcam, ab110413, 1:1000), rabbit anti-PGC-1α (Millipore, ST1204, 1:1000), rabbit anti-TFAM (Abcam, ab131607, 1:1000), rabbit anti-IBA1 (Cell signaling, 17198, 1:1000), mouse anti-GFAP (Millipore, MAB360, 1:1000), mouse anti-CNPase (Millipore, MAB326, 1:500), rabbit anti-MBP (Millipore, AB980, 1:2000) and mouse anti-Actin (Millipore, MAB1501, 1:10,000). HRP-conjugated anti-rabbit and anti-mouse secondary antibodies (Vector Laboratories, Burlingame, CA) were then applied. The signal was visualized by Pierce SuperSignal Chemiluminescent Substrates or SuperSignal West Pico Chemiluminescent Substrate (Thermo Scientific, IL) and captured by ChemiDoc MP Imaging System (BioRad, Hercules, CA). Positions of molecular weight markers (kDa) were indicated. All band intensities were quantified using Image Lab 6.0.1 (Bio-Rad, Hercules, CA) or ImageJ and normalized to corresponding Actin intensity.

### 2.10 Immunohistochemistry

Animals were transcardially perfused with phosphate-buffered saline (PBS) for 10 min. The brains were immersion fixed with intact skull with 4% paraformaldehyde for 48 h, then washed with PBS and stored in PBS/0.01% Azide at 4°C until sectioned. Brains were then harvested from the skull and soaked in 30% sucrose solution (in PBS) for 1 day. The brains were embedded in gelatin using the MultiBrain Technology (Neuroscience Associates, Knoxville, TN) and were coronally sectioned into 40 μm thick sections. The sections were incubated with rabbit anti-IBA1 (FujiFilm Wako Pure Chemical corporation, 019-19741, 1:500) and rabbit anti-MBP (Cell Signaling 78896s, 1:250) overnight followed by Alexa Fluor 488 or 555 goat anti-rabbit secondary antibodies for 60 min at room temperature. Myelin was labeled with FluoroMyelin™ Green (Invitrogen B34650, 1:300). Nuclei were stained with DAPI. Images were acquired with Zeiss LSM 880 Airyscan Confocal Microscope at 20X magnification for IBA1 and MBP staining, and at 10X magnification for myelin labeling. Images were analyzed with Imaris 10.0 software.

### 2.11 RNA Isolation

Frozen hippocampal tissues were directly homogenized in TRIzol^®^ Reagent, followed by chloroform extraction at a volume ratio of 1:5 to that of the TRIzol^®^ Reagent. Ethanol was then used to precipitate nucleic acids from the aqueous phase. RNA was further purified using PureLink RNA Mini Kit (Invitrogen, 12183018A) following manufacturer’s instructions. Purelink DNase (Invitrogen, 12185010) was used to eliminate DNA contamination. Purified RNA was eluted in RNase-free diH_2_O. RNA concentration and quality were checked by NanoDrop™ One.

### 2.12 Real-time quantitative PCR

Purified RNA was then reverse transcribed to cDNA using SuperScript VILO Master Mix (Invitrogen, 11755250). 30ng cDNA was used with TaqMan Universal PCR Master Mix (Applied Biosystems, 4304437). The following TaqMan primers were used: *Aif1* Mm00479862_g1; *Ppargc1a* Mm01208835_m1; *Nrf1* Mm00447996_m1 and *Actb* Mm00607939_s1. Target cDNA was amplified and detected using Applied Biosystems QuantStudio 6 Flex system. Relative gene expression level (fold change) to reference group was calculated by the comparative Ct (ΔΔCt) method (Schmittgen and Livak, 2008).

### 2.13 RNA-sequencing (RNA-Seq)

RNA-Seq was conducted using hippocampal RNA samples at Vanderbilt Technologies for Advanced Genomics (VANTAGE). Only RNA samples with an acceptable RNA quality indicator score (RQI > 7) were used for sequencing. mRNA enrichment and cDNA library preparation were done using a stranded mRNA (poly(A) - selected) sample preparation kit. Sequencing was performed at 150bp paired-end on NovaSeq6000, targeting 50 million reads per sample. Transcripts were mapped to Mouse cDNA (ensembl release 95) using Salmon (version 0.15.0) (Patro et al., 2017). Tximport (version 1.24.0) (Soneson et al., 2015) was used to generate a counts table from Salmon output, and DESeq2 (version 1.36.0) (Love et al., 2014) was used to calculate normalized read counts for each gene to perform expression analysis. The RNA-seq data included 3–7 animals per group. Statistical significance for RNA-seq data was calculated by DESeq2 V1.36.0.

To visualize the expression patterns of selected genes across samples, a heatmap was generated using the *pheatmap* R package (v1.0.12). The analysis utilized normalized count data that was transformed using the variance-stabilizing transformation (vst from *DESeq2* (Love et al., 2014) R package (v1.36.0) to produce expression values suitable for visualization. For the heatmap, these expression values were averaged within each group and then scaled on a per-gene basis, the resulting z-scores are presented.

Volcano plots were generated to visualize differential gene expression within selected pathways. For each comparison, the log2Fold Change (derived from DESeq2 analysis) was plotted against the negative log10 of the *p*-value. Genes identified as statistically significant (*p*-value < 0.05) and belonging to the selected pathways were highlighted: upregulated genes were colored red, and downregulated genes were colored green. A dashed horizontal line indicated the *p*-value threshold of 0.05.

### 2.14 Ingenuity pathway analysis (IPA)

RNA-seq data was processed by the core analysis function of IPA using a *p* value cutoff of 0.05 (Wang T. et al., 2023). The canonical pathways are identified based on enrichment of qualified genes. The upstream regulator analysis predicted activation or inhibition of regulatory molecules based on expression of respective downstream genes and networks compiled from literature and IPA’s Ingenuity knowledge base.

### 2.15 Eigengene expression score

Eigengenes were calculated following the method previously described (Alter et al., 2000) to summarize the expression profiles of a given list of genes into a single representative measure. This process utilized singular value decomposition (SVD), a mathematical approach that decomposes a matrix into orthogonal components. Specifically, the scaled and variance-stabilized transformation of the normalized expression counts was used as input for the SVD. The first singular vector, which captures the majority of the variance in the dataset, was extracted as the eigengene for each sample within the gene list. To maintain biological interpretability, the sign of the eigengene was standardized: if the correlation between the eigengene and the average expression of the gene set was negative, the signs of the eigengene values were flipped. This approach ensures that the eigengene accurately reflects the overall expression pattern of the given genes across samples, providing a robust metric for downstream analysis.

### 2.16 Mitochondrial respiration in frozen samples

Mitochondrial respiration in frozen samples was assessed as previously described (Acin-Perez et al., 2020). Briefly, frozen cortical tissue was homogenized with a plastic pestle homogenizer in MAS buffer (70 mM Sucrose, 220 mM Mannitol, 5 mM KH2PO4, 5 mM MgCl2, 1 mM EGTA, 2 mM HEPES, pH 7.4 adjusted with KOH) and centrifuged at 1000x*g* for 10 min at 4°C. The supernatant was transferred to a 1.5 mL microcentrifuge tube and protein content was measured with a BCA protein assay kit (Pierce, Rockford, IL). A total of 4 μg protein was loaded per well in a volume of 20 μL and the plate was centrifuged at 2000x*g* for 5 min at 4°C (no brake). After centrifugation, the final volume was brought to 150 μL with MAS buffer supplemented with 10 μg/mL of both cytochrome c (Sigma C2506) and alamethicin (Sigma A4665) and the plate was loaded into the XF96 Extracellular Flux Analyzer. Substrate injection was as follows: NADH (1 mM, Sigma N4505) was injected at port A; rotenone (4 μM, Sigma R8875) + antimycin (4 μM, Enzo life Sciences 380-075-M010) at port B; TMPD (0.5 mM, Sigma 87890) + ascorbic acid (1 mM, Fisher A61-100) at port C; and azide (50 mM, Sigma S8032) at port D.

### 2.17 Statistical analysis

All bar graphs were presented as means ± standard error of the mean (S.E.M.), with individual data points plotted. For all bar graphs involving multiple comparisons, statistical analyses were performed between APOE3 and APOE4 within the same chronological and endocrinological groups, as well as between different chronological and endocrinological groups within each genotype, using multiple comparison test. Specifically, statistically significant differences across all chronological and endocrinological aging (CEA) and genotype groups were determined using two-way ANOVA, followed by Tukey’s *post hoc* test. This approach incorporated both chronological age and cycling status to capture both endocrine and chronological aging processes, as established in our previous publications (Mishra et al., 2020, Wang et al., 2020b,Yin et al., 2015, Klosinski et al., 2015). To further validate our findings, we applied a nested model in which cycling status was nested within age: *Outcome* ∼ *Genotype* × *Age* + *Genotype* × *Cycling_nested*. Here, *Cycling_nested* denotes composite levels combining age and cycling status (e.g., “9M_Irreg”), to account for the age-specific availability of cycling levels. Type III ANOVA was performed using sum-to-zero contrasts to estimate main effects and interactions. The results (Supplementary Table 1) were consistent with those observed from the two-way ANOVA, with age and cycling status exhibiting more pronounced effects, further supporting our original conclusions.

Statistically significant differences between different menopausal and genotype groups with same age were determined using two-way ANOVA with Holm-Sidak multiple comparisons test.

Two-way ANOVA analyses were conducted using GraphPad Prism with Type III sums of squares. Sum contrasts were applied to all categorical factors to ensure orthogonality of the model terms. The result of two-way ANOVA analysis was displayed in the corner text for each bar chart. For all results showing significant differences by two-way ANOVA, effect sizes, reported as Partial Eta-squared (ηp^2^), were calculated and presented in Supplementary Table 2. Statistically significant differences between two groups were determined using unpaired *t*-test. Comparisons with an adjusted *p* value smaller than 0.05 were considered statistically significant.

### 2.18 UK biobank data analysis

#### 2.18.1 Data source

This research has been conducted using the UK Biobank (UKB) Resource under Application Number 72504 “Identification of precision therapeutics for Alzheimer’s disease prevention and treatment.” UKB received ethical approval from the National Health Service (NHS) Northwest Centre for Research Ethics Committee. The UKB is a large-scale biomedical database and research resource, encompassing data from approximately half a million participants aged 40–69 years, with a balanced representation of women and men. Recruitment occurred between 2006 and 2010 across 33 centers throughout the UK, ensuring diverse socioeconomic, ethnic, and urban–rural representation (UK Biobank, 2023). At baseline, participants provided information regarding their socio-demographic, lifestyle, environmental, and health-related attributes through touchscreen questionnaires and an interview conducted by a nurse. Participants underwent physical assessments and blood samples were collected. All participants provided electronic signed consent for their data to be used in health-related research. The exposures of interest in this study were age of menopause and APOE genotype.

#### 2.18.2 Study design and variables

This study included female participants from UKB with complete data on APOE genotype and age at menopause. AD diagnosis was determined using the International Classification of Diseases, Tenth Revision, Clinical Modification (ICD-10-CM) codes from primary care (UKB Data Field number: 41202) aggregated into single-level categories (Dagliati et al., 2021, Torrandell-Haro et al., 2020). Age at menopause was defined as the highest reported menopause age across four UK Biobank assessment visits. Age at menopause was categorized as ≤45 years (early), and >45 years (regular). Level of education was categorized as low or high and cardiovascular disease (CVD) history was coded as a binary variable (yes/no). The primary outcome variable in this study was the first occurrence of AD.

Genetic information of single-nucleotide polymorphisms (SNPs) was available for a subset of participants (*N* = 299,627) through genotyping arrays and imputation methods (Bycroft et al., 2018). Genotyping arrays included the UK BiLEVE Axiom array and the UKB Axiom array for direct SNP genotyping with imputed genotypes derived from the Haplotype Reference Consortium and UK10K haplotype resource using UKB’s computational pipeline. Genetic analysis for this study employed version 3, published in March 2018, with genotyping quality control centrally carried out by the UKB (Loh et al., 2018). For identification of APOE genotype, we focused on the two APOE-related key SNPs, rs429358 and rs7412. APOE4 carriers possess at least one ε4 allele. Therefore, participants with genotypes ε2/ε4, ε3/ε4, or ε4/ε4 were classified as APOE4 carriers. Conversely, individuals with genotypes ε2/ε2, ε2/ε3, or ε3/ε3 do not carry the ε4 allele and were categorized as APOE4 non-carriers. APOE genotype information was available for a total of 248,056 participants.

#### 2.18.3 Statistical analysis

The frequency (percentage) of participants with and without an AD diagnosis was calculated for categorical variables, while mean (standard deviation) was reported for continuous variables. Group differences were assessed using Pearson’s chi-squared test or Fisher’s exact test (for expected values <5) for categorical variables and analysis of variance (ANOVA) for continuous variables. Statistical significance was set at a two-sided *p*-value < 0.01.

A logistic regression model was employed to assess the effect of APOE genotype on age at menopause. Odds ratios (OR) and 95% confidence intervals (CI) were reported to quantify the relative risk of APOE genotype on age of menopause. Regular menopause age was defined as reference.

A logistic regression model was employed to assess the combined effects of APOE genotype and age at menopause on AD risk. An interaction term was included to evaluate statistical interactions between these variables. OR and 95% CI were reported to quantify the relative risk of AD across different levels of APOE genotype and age of menopause. APOE4 non-carrier with regular menopause age was defined as reference. To reduce confounder effects, we excluded female participants with history of hormone replacement therapy, and we controlled for confounders significantly different between the AD and non-AD group, including level of education and CVD history.

## 3 Results

### 3.1 Accelerated endocrine aging in APOE4 females

To determine the impact of APOE genotype on midlife endocrine aging, the endocrine status and plasma hormone profiles were characterized in female APOE3 and APOE4 mice. While perimenopause is a staged continuum, 6-, 9-, and 15-month-old animals were analyzed to assess the effect of the APOE genotype across the perimenopause to menopause transition and thus included young (regularly cycling, 6M-Reg), early perimenopausal transition (regular to irregular cycling, 9M-Reg and 9M-Irreg), and late perimenopausal transition (irregular cycling to acyclicity, 15M-Irreg and 15M-Acyc), respectively.

**FIGURE 1:**
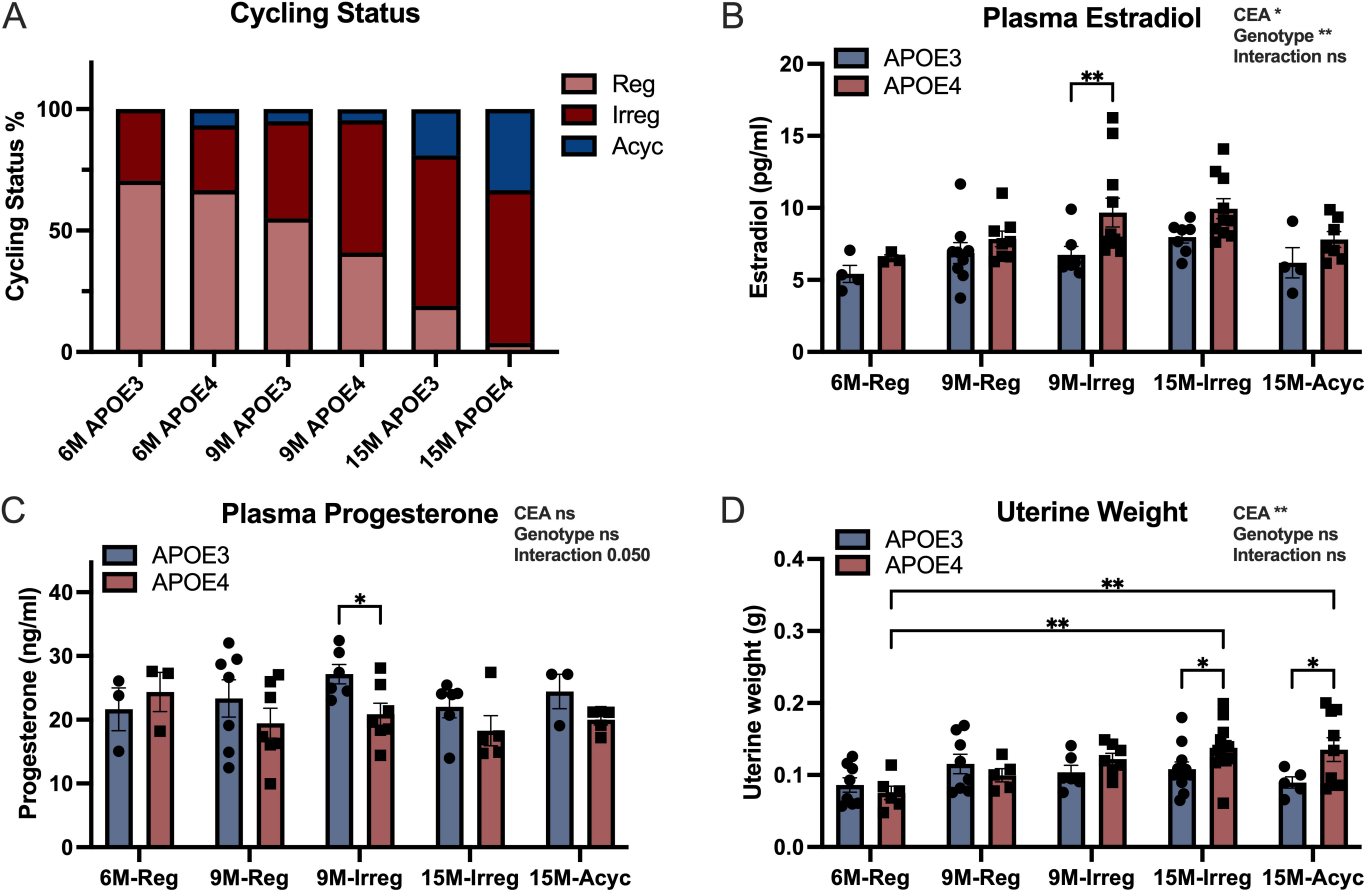
Accelerated endocrine aging in APOE4 females. **(A)** Percentage of APOE3 and APOE4 females by cycling status (total *n* = 122, 17 APOE3 6M, 20 APOE3 9M, 21 APOE3 15M, 15 APOE4 6M, 22 APOE4 9M, and 27 APOE4 15M). Plasma levels of estradiol (**B**, *n* = 3–11/group) and progesterone (**C**, *n* = 3–7/group) in all groups. **(D)** Uterine weights of the animals (*n* = 5–13/group). Data were presented as Mean ± SEM with individual data plotted. Statistically significant differences were determined using two-way ANOVA, followed by Tukey′s *post hoc* test. *p < 0.05, **p < 0.01. CEA, chronological and endocrinological aging.

Outcomes of these analyses indicated that APOE4 females exhibited accelerated endocrine aging with a greater percentage advancing to perimenopause and menopause relative to their APOE3 counterparts (Figure 1A). At 6 months, APOE3 and APOE4 females exhibited a similar percent of regular and irregular cyclers. Surprisingly, acyclicity emerged in a small percentage of APOE4 females at 6 months. The trend toward accelerated endocrine aging was evident at 9 months when APOE4 females exhibited a lower percent of regular cyclers with a concomitant rise in the percent of irregular cyclers, compared to APOE3 females. At 15 months, APOE4 females were characterized by a preponderance of irregular cyclers and acyclic cyclers relative to APOE3 females.

Consistent with an accelerated perimenopausal profile in APOE4 females, APOE4 9M-Irreg group exhibited significantly higher plasma estradiol (E2, Figure 1B) and lower progesterone levels (P4, Figure 1C) compared to their APOE3 counterparts. Consistent with higher plasma estradiol, APOE4 15M-Irreg and 15M-Acyc groups exhibited a significant increase in uterine weight compared to APOE4 6M-Reg group and APOE3 15M counterparts (Figure 1D). The rise in plasma estradiol at 9 months and increase in uterine weight at 15 months are consistent with the accelerated loss of cyclicity in the APOE4 females. Collectively, these results indicated accelerated endocrine aging of APOE4 females.

### 3.2 Shift in peripheral metabolic phenotype in APOE4 PAM females

To further assess the impact of APOE genotype on female midlife metabolic aging, body weight and composition and levels of peripheral metabolic indicators were determined in all groups.

**FIGURE 2:**
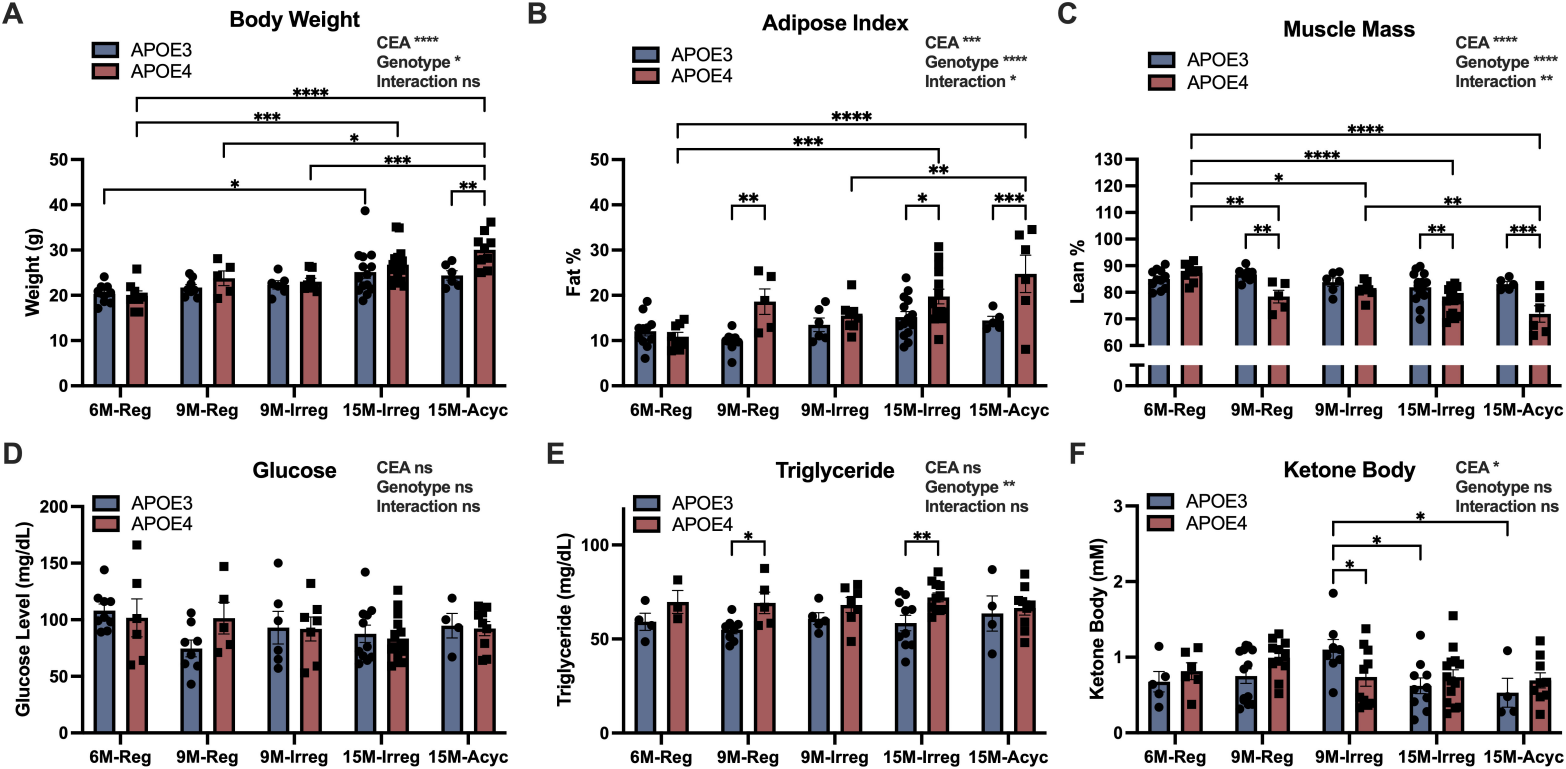
Shift in peripheral metabolism in APOE4 PAM females. Body weight **(A)**, adipose composition **(B)** and muscle composition **(C)** of APOE3 and APOE4 females with different age and endocrine status (*n* = 5–17/group). Fasting levels of glucose **(D)**, triglyceride **(E)** and ketone bodies **(F)** in all groups (*n* = 3–14/group). Data were presented as Mean ± SEM with individual data plotted. Statistically significant differences were determined using two-way ANOVA, followed by Tukey′s *post hoc* test. *p < 0.05, **p < 0.01, ***p < 0.001, ****p < 0.0001. CEA, chronological and endocrinological aging.

While both APOE3 and APOE4 females exhibited increased body weight with chronological and endocrinological aging (Figure 2A), APOE4 females exhibited a significant shift in body composition profile with aging compared to APOE3 counterparts (Figures 2B, C). Significantly higher adipose composition and lower muscle composition were observed in APOE4 9M and 15M groups compared to APOE4 6M-Reg group and APOE3 counterparts (Figures 2B, C). Consistently, APOE4 females exhibited stable higher plasma triglyceride level compared to APOE3 counterparts (Figure 2E), consistent with our previous findings (Shang et al., 2020). In contrast, no genotype effects were observed on glucose levels (Figure 2D), suggesting a greater impact of APOE4 genotype on lipid metabolism. Consistent with accelerated endocrine aging, menopause-induced decrease in plasma ketone body levels emerged earlier in APOE4 females, evidenced by significantly reduced ketone body levels in APOE4 9M-Irreg group relative to APOE3 counterparts (Figure 2F).

Collectively, the peripheral metabolic profile indicated that APOE4 females exhibited accumulation of adipose tissue with high plasma triglyceride and accelerated ketone body decline during menopausal transition, suggestive of a shift in metabolic phenotype in these animals.

### 3.3 Deficits in metabolic reprogramming in APOE4 PAM brains

The phenotypic data indicating accelerated endocrine aging in the periphery of APOE4 female mice led to analyses to determine the impact of endocrine aging and APOE4 genotype on key metabolic pathways in brain.

**FIGURE 3:**
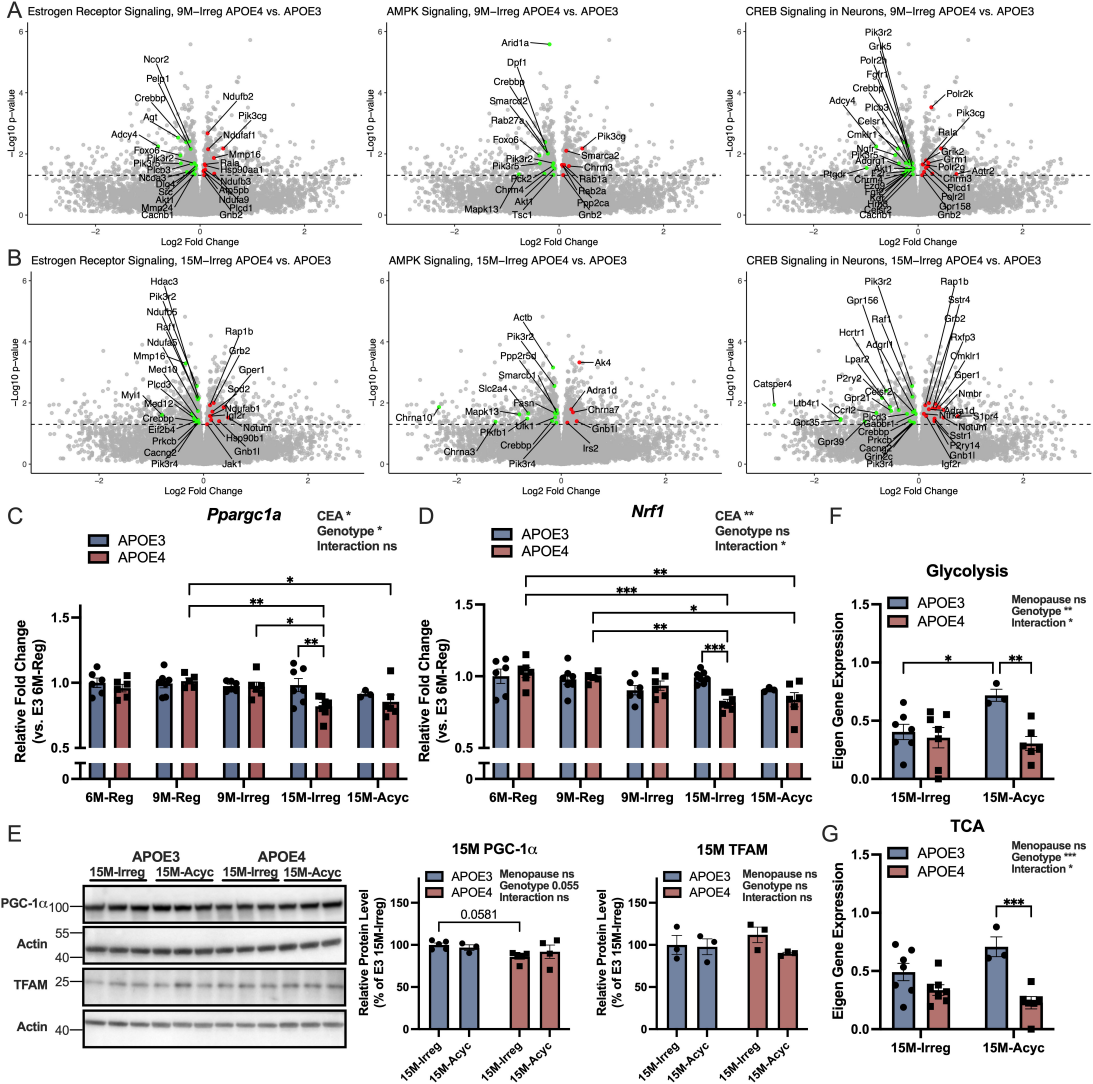
Deficits in upstream regulators required for brain metabolic reprogramming in APOE4 PAM brains. **(A)** Volcano plots showing differentially expressed genes involved in estrogen receptor, AMPK and CREB signaling pathways in the 9M-Irreg APOE4 group compared to the APOE3 group, with the corresponding gene list provided in Table 1. **(B)** Volcano plots showing differentially expressed genes involved in estrogen receptor, AMPK and CREB signaling pathways in the 15M-Irreg APOE4 group compared to the APOE3 group, with the corresponding gene list provided in Table 1. Hippocampal expression levels of *Ppargc1a* **(C)** and *Nrf1* **(D)** of APOE3 and APOE4 mice (*n* = 3–7). **(E)** Cortical levels of PGC-1α and TFAM of 15M-Irreg and 15M-Acyc APOE3 and APOE4 mice (*n* = 3–5). Pathway-level hippocampal gene expression of glycolysis **(F)** and TCA cycle **(G)** of 15M-Irreg and 15M-Acyc APOE3 and APOE4 mice (*n* = 3–7). Data were presented as Mean ± SEM with individual data plotted. **(C,D)** Statistically significant differences were determined using two-way ANOVA, followed by Tukey′s *post hoc* test. (**E–G)** Statistically significant differences were determined using two-way ANOVA with Holm-Sidak multiple comparisons test. *p < 0.05, **p < 0.01, ***p < 0.001. CEA: chronological and endocrinological aging.

Transcriptomic analysis (Figures 3A, B and Table 1) using IPA indicated reduced estrogen receptor signaling in APOE4 9M-Irreg and 15M-Irreg groups relative to APOE3 counterparts consistent with accelerated dismantling of estrogen signaling in APOE4 PAM females. Estrogen is a master regulator of female metabolism (Rettberg et al., 2014), modulating several key metabolic regulators, including AMP-activated protein kinase (AMPK), peroxisome proliferator-activated receptor gamma coactivator 1α (*Ppargc1a*/PGC-1α) and nuclear respiratory factor-1 (NRF1) (Willy et al., 2004, Gaignard et al., 2017, Marin et al., 2017). Consistent with decreased estrogen receptor signaling, AMPK signaling pathway was downregulated in APOE4 9M-Irreg and 15M-Irreg groups, which was accompanied with decreased CREB (cAMP-response element binding protein) signaling (Figures 3A, B and Table 1). Further, APOE4 females exhibited significantly reduced transcriptomic levels of *Ppargc1a/*PGC-1α (Figure 3C) and *Nrf1* (Figure 3D) with aging, resulting in significantly lower levels of *Ppargc1a* and *Nrf1* in APOE4 15M-Irreg group compared to the APOE3 counterparts. Consistently, a trend toward decreased PGC-1α protein expression was also observed in APOE4 15M-Irreg group relative to APOE3 15M-Irreg group (Figure 3E). In contrast, TFAM (mitochondrial transcription factor A) protein level was not different across 15M APOE3 and APOE4 groups. These results are predictive of inhibited AMPK-PGC-1α-NRF1 signaling during the perimenopausal transition in APOE4 female brains. Further, an increase in pathway-level expression of genes involved in glycolysis (Figure 3F) and the TCA cycle (Figure 3G) occurred in APOE3 15M-Acyc group, compared to APOE3 15M-Irreg group. In contrast, this increase in gene expression involved in glycolysis and the TCA cycle did not occur in APOE4 15M-Acyc group, resulting in significantly lower pathway-level gene expression of glycolysis and the TCA cycle in APOE4 15M-Acyc group, compared to APOE3 counterparts.

**TABLE 1:** Reduced estrogen receptor, AMPK, and CREB signaling in APOE4 groups.

	Pathway	Z-score	*P*-Value	Genes (Red indicates upregulation, green indicates down-regulation)
9M-Irreg APOE4 vs. APOE3	Estrogen receptor signaling	−1.147	1.31E-02	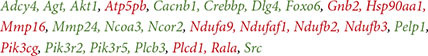
	AMPK signaling	−1.155	1.84E-03	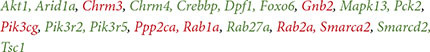
	CREB signaling in neurons	−2.414	2.96E-02	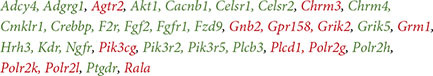
15M-Irreg APOE4 vs. APOE3	Estrogen receptor signaling	−0.688	1.95E-02	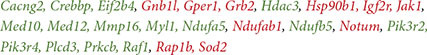
	AMPK signaling	−0.577	7.79E-03	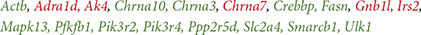
	CREB signaling in neurons	−1.029	9.68E-03	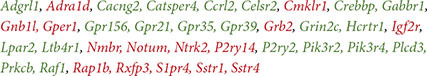

Collectively, these results indicate an accelerated loss of estrogen signaling and downregulation of the AMPK-PGC-1α-NRF1 pathway which was associated with an inability to mount an adaptive metabolic reprogramming response in the APOE4 female PAM brain.

### 3.4 APOE4 PAM females exhibited greater mitochondrial deficits

**FIGURE 4:**
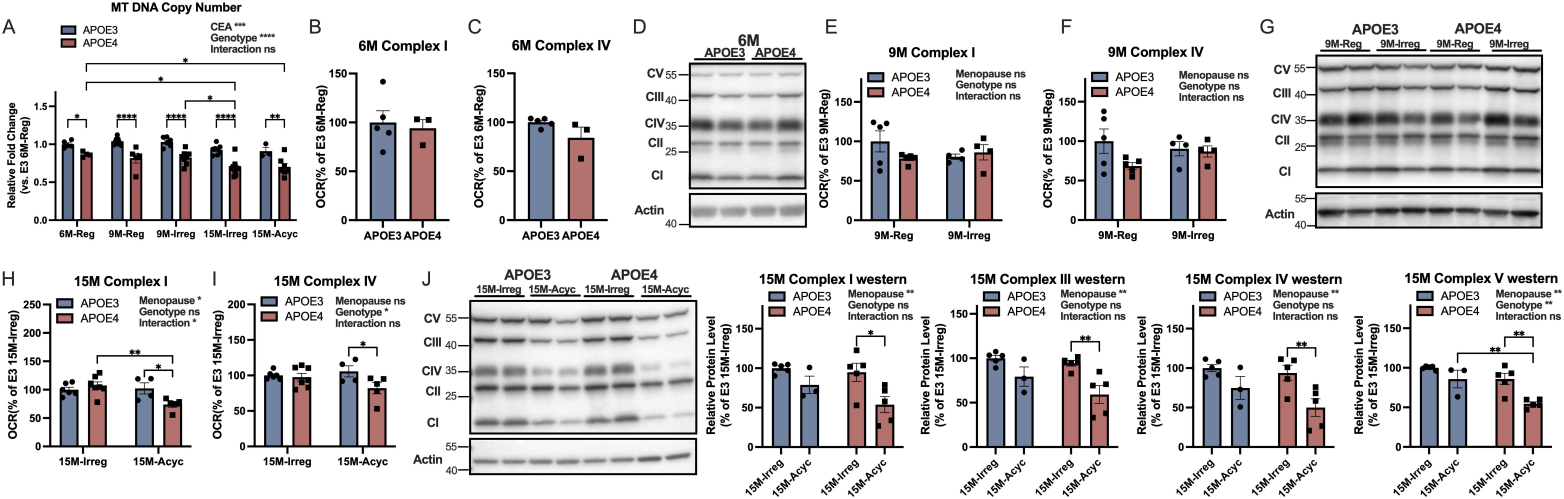
APOE4 perimenopausal brain exhibited greater mitochondrial deficits. **(A)** Mitochondrial DNA copy number in all groups (*n* = 3–9). Statistically significant differences were determined using two-way ANOVA, followed by Tukey′s *post hoc* test. Cortical complex I **(B)** and IV **(C)** activities and complex protein levels **(D)** of 6M-Reg APOE3 and APOE4 females (*n* = 3–5/group). Cortical complex I **(E)** and IV **(F)** activities and complex protein levels **(G)** of 9M-Reg and 9M-Irreg APOE3 and APOE4 females (*n* = 4–5/group). At 15 months, cortical complex I **(H)** and IV **(I)** activities (*n* = 4–7/group) and complex protein levels (**J**, *n* = 3–5/group) were significantly reduced in APOE4 15M-Acyc groups. Data were presented as Mean ± SEM with individual data plotted. (**E**–**J)** Statistically significant differences were determined using two-way ANOVA with Holm-Sidak multiple comparisons test. *p < 0.05, **p < 0.01, ***p < 0.001, ****p < 0.0001. CEA: chronological and endocrinological aging.

Consistent with accelerated dismantling of the estrogen bioenergetic system in brain, compromised mitochondrial function was observed in APOE4 PAM females. Mitochondrial DNA copy number was significantly lower in APOE4 groups compared to APOE3 counterparts which was evident across all 5 chronological and endocrinological aging stages with a more profound decrease with aging in APOE4 15M groups (Figure 4A). While no significant differences in cortical complex I and IV activities and protein levels were observed between genotypes at 6 months (Figures 4B–D) and 9 months of age (Figures 4E–G), at 15 months of age, significant decreases in complex I (Figure 4H) and complex IV activities (Figure 4I) occurred in APOE4 15M-Acyc groups compared to APOE3 15M-Acyc groups. The decreased mitochondrial function was accompanied by significantly reduced complex I, III, IV, and V (Figure 4J) protein levels in APOE4 15M-Acyc groups.

Collectively, these outcomes demonstrated that the bioenergetic crisis in brain was greater in the APOE4 postmenopausal female brain.

### 3.5 Increased microglial activation parallels metabolic phenotype changes in APOE4 PAM females

Inflammation is correlated with menopause-induced metabolic shift (Mishra and Brinton, 2018, Mishra et al., 2020). Therefore, the accelerated dismantling of estrogen signaling and greater bioenergetic deficits in APOE4 PAM females could lead to enhanced activation of neuroimmune phenotype. To test this hypothesis, brain *Aif1/*IBA1 (ionized calcium-binding adaptor molecule 1) RNA and protein levels and inflammatory factor levels were assessed.

**FIGURE 5:**
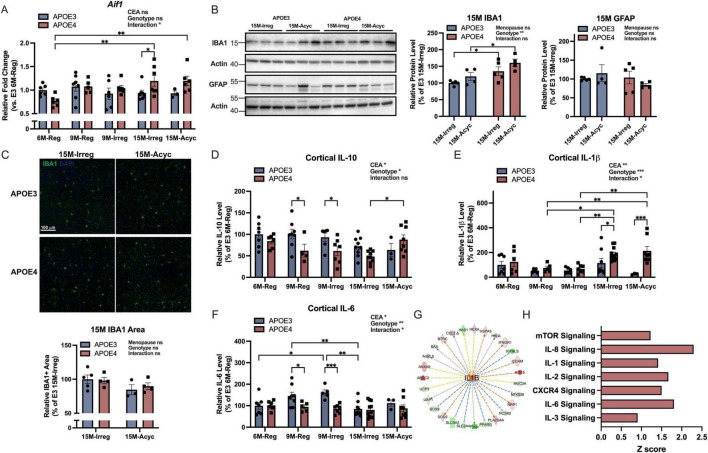
Increased microglial activation in APOE4 PAM brains. **(A)** *Aif1* RNA levels in all groups (*n* = 3–7). **(B)** Protein levels of IBA1 and GFAP of 15M-Irreg and 15M-Acyc APOE3 and APOE4 groups (*n* = 4–5/group). The actin control for IBA1 is the same as the actin control used for TFAM in Figure 3E. **(C)** Anti-IBA1 staining on brain sections from 15M-Irreg and 15M-Acyc APOE3 and APOE4 groups (*n* = 3–5, Bregma –2.2 mm). Cortical levels of IL-10 **(D)**, IL-1β **(E)** and IL-6 **(F)** in all groups (*n* = 3–11). **(G)** IL1B was predicted to be activated in APOE4 15M-Irreg group compared to APOE3 counterparts. **(H)** IPA canonical pathways indicated activation of multiple cytokine, mTOR and CXCR4 signaling pathways in APOE4 15M-Acyc group compared to APOE3 counterparts. Data were presented as Mean ± SEM with individual data plotted. (**A, D–F)** Statistically significant differences were determined using two-way ANOVA, followed by Tukey′s *post hoc* test. **(B,C)** Statistically significant differences were determined using two-way ANOVA with Holm-Sidak multiple comparisons test. *p < 0.05, **p < 0.01, ***p < 0.001. CEA: chronological and endocrinological aging.

As shown in Figure 5A, *Aif1/*IBA1 RNA levels were significantly increased in APOE4 15M groups compared to APOE4 6M-Reg group, resulting in significant higher levels of *Aif1* in APOE4 15M-Irreg group compared to APOE3 counterparts. Consistently, significantly higher IBA1 protein levels were observed in both APOE4 15M-Irreg and 15M-Acyc groups compared to APOE3 counterparts (Figure 5B), although cortical IBA1 + area did not differ significantly between APOE3 and APOE4 15M groups (Figure 5C). In contrast, glial fibrillary acidic protein (GFAP) protein levels were not different between genotypes nor across late perimenopausal transition (Figure 5B), confirming APOE4-specific microglial activation in PAM females. Consistently, compared to APOE3 counterparts, significantly lower anti-inflammatory IL-10 levels were observed in APOE4 9M-Reg and 9M-Irreg groups (Figure 5D), accompanied with a significant and persistent increase in pro-inflammatory IL-1β levels in APOE4 15M-Irreg and 15M-Acyc groups (Figure 5E), relative to APOE4 9M groups and APOE3 counterparts. In contrast, APOE3 females exhibited a transient increase in cortical IL-6 levels at 9M, which significantly decreased in APOE3 15M-Irreg group (Figure 5F). Bulk RNA-seq analysis (IPA) outcomes confirmed the upregulation of IL1B signaling in APOE4 15M-Irreg group (Figure 5G) which was predictive of activation of mTOR, IL-8, IL-1, IL-2, CXCR4, IL-6 and IL-3 signaling pathways in APOE4 15M-Acyc group, compared to APOE3 counterparts (Figure 5H).

Together with accelerated endocrine aging and metabolic dysfunction, APOE4 females also exhibited a pro-inflammatory phenotype compared to APOE3 counterparts.

### 3.6 Alteration in myelination in APOE4 PAM brains

**FIGURE 6:**
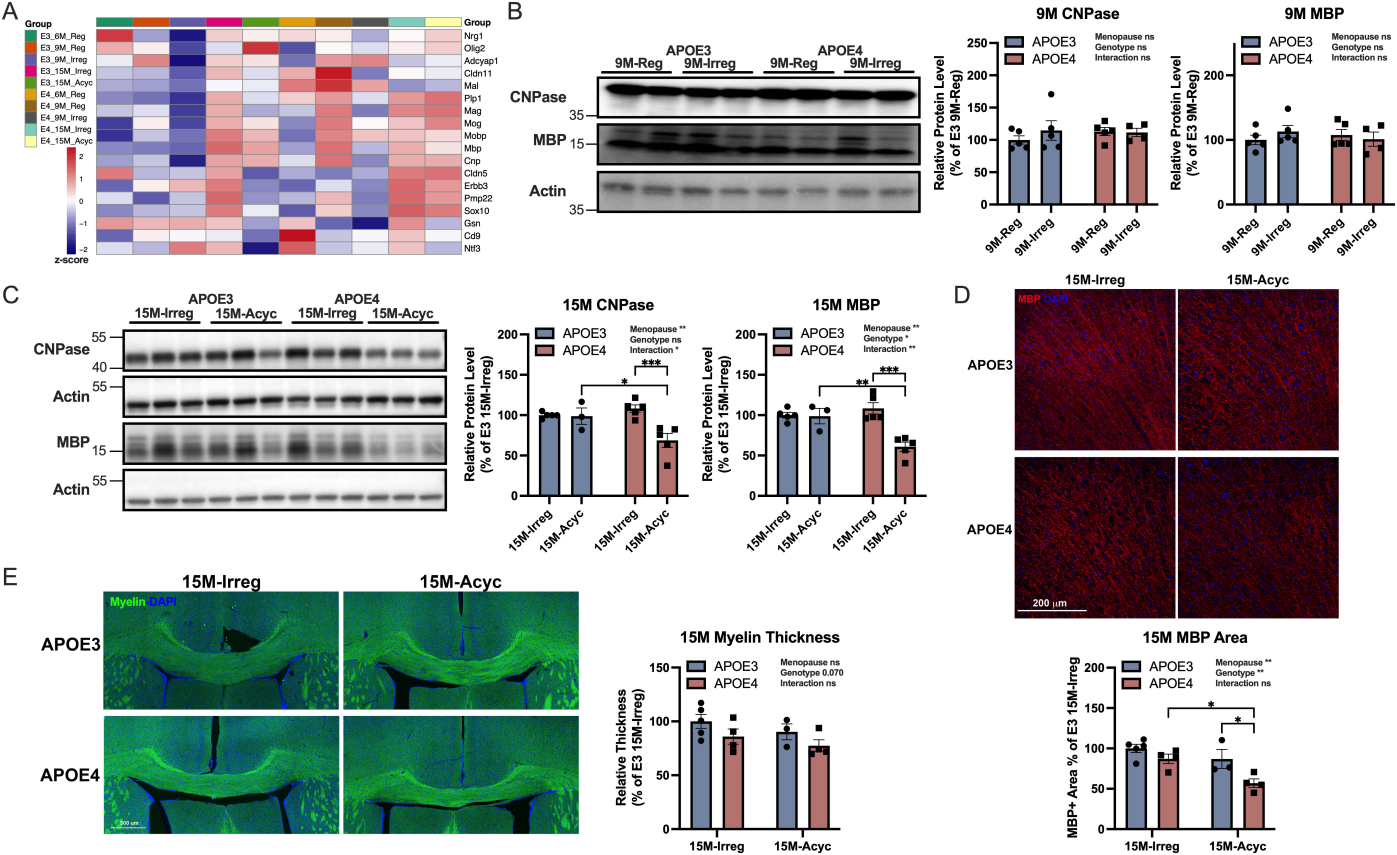
Increased demyelination in APOE4 PAM brains. **(A)** Myelin metabolic and generation related gene expression in all groups (z-score). **(B)** CNPase and MBP protein levels in APOE3 and APOE4 9M groups (*n* = 4–5). **(C)** CNPase and MBP protein levels in APOE3 and APOE4 15M groups (*n* = 3–5). **(D)** Anti-MBP staining on brain sections from 15M-Irreg and 15M-Acyc APOE3 and APOE4 females (*n* = 3–5, Bregma –2.2 mm). **(E)** Corpus callosum thickness in APOE3 and APOE4 15M groups (*n* = 3–5, Bregma 0.2 mm). Data were presented as Mean ± SEM with individual data plotted. Statistically significant differences were determined using two-way ANOVA with Holm-Sidak multiple comparisons test. *p < 0.05, **p < 0.01, ***p < 0.001.

During female brain aging, bioenergetic dysfunction can lead to white matter degeneration (Klosinski et al., 2015, Mosconi et al., 2017b), which could be exacerbated by APOE4 genotype (Blanchard et al., 2022). Hence, the impact of APOE genotype and endocrine aging on brain white matter integrity was investigated. Consistent with observations in the perimenopausal wild-type mouse model (Klosinski et al., 2015), the expression of genes involved in myelin generation and repair were upregulated in APOE3 15M-Irreg group and decreased in APOE3 15M-Acyc group (Figure 6A). In contrast, the upregulation of myelin-related genes was observed earlier in APOE4 9M-Reg group, consistent with accelerated aging, and remained high in APOE4 15M-Irreg and 15M-Acyc groups.

CNPase (2′, 3′-cyclic-nucleotide 3′-phosphodiesterase, a marker for oligodendrocytes) and MBP (myelin basic protein) protein expression did not change with early endocrine aging at 9 months (Figure 6B), whereas at 15 months, a significant decline in CNPase and MBP protein levels was evident in the APOE4 15M-Acyc group compared to APOE4 15M-Irreg group and APOE3 15M-Acyc groups (Figure 6C). Consistently, MBP + cortical area was significantly reduced in APOE4 15-Acyc group compared to APOE3 counterparts (Figure 6D). Further, a trend toward reduced corpus callosum thickness in 15M APOE4 groups was observed, compared to the APOE3 counterparts (Figure 6E). Together, these results indicate that changes in indicators of white matter integrity during menopausal transition were more pronounced in APOE4 carriers.

### 3.7 APOE4 is associated with early menopause in women

**FIGURE 7:**
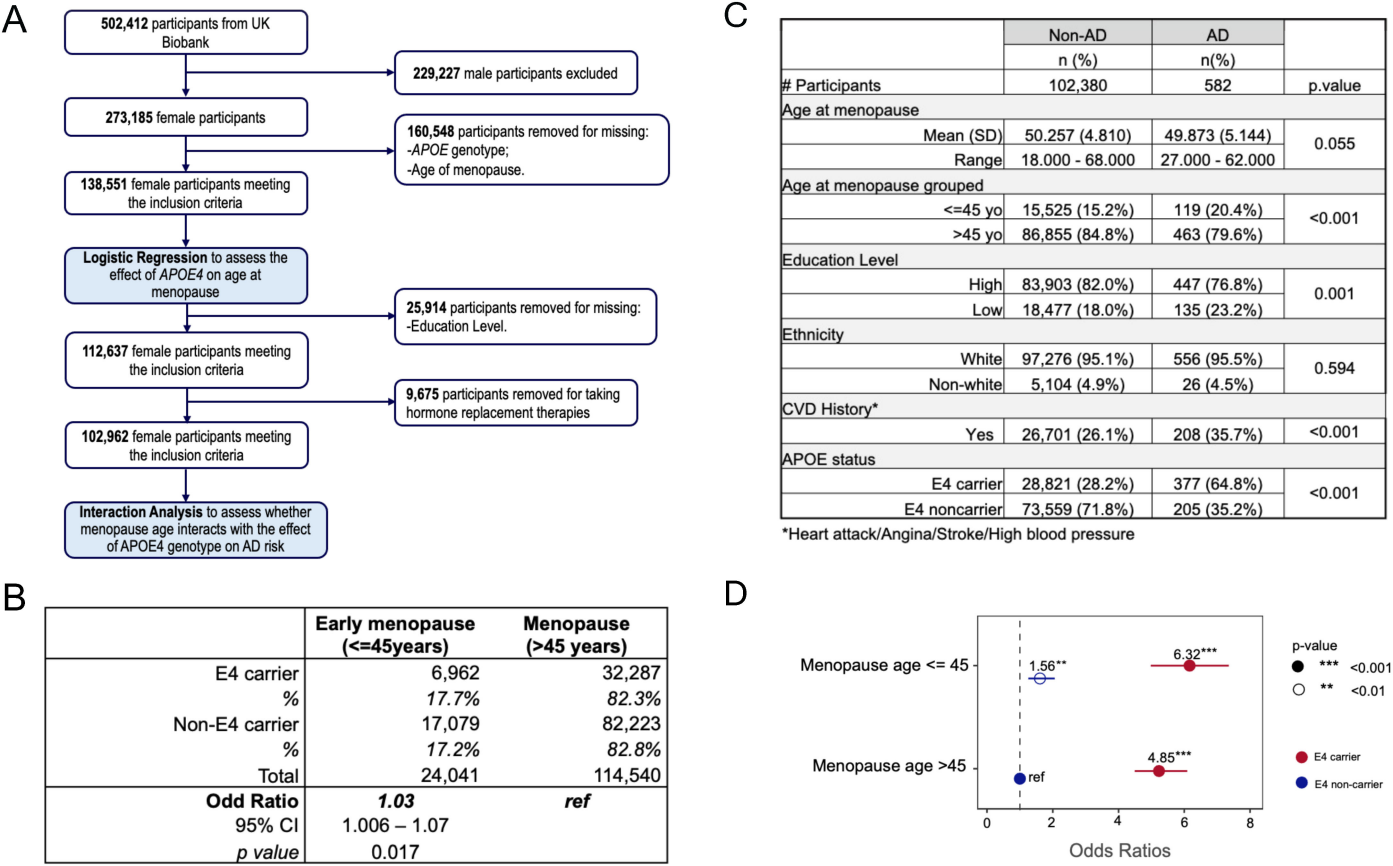
APOE4 is associated with early menopause in women. **(A)** Study design. **(B)** APOE4 is associated with early menopause age. **(C)** Participant characteristics. **(D)** Odds ratio of AD risk for APOE4 carriers and non-carriers with early or normal menopausal age. **p < 0.01, ***p < 0.001.

To investigate the translational validity of the accelerated aging observed in our APOE4 PAM models, we analyzed the effect of APOE4 on age of menopause in women using UKB. Of the 273,185 women within the UKB, 138,551 reported natural menopause and APOE4 genotype (Figure 7A). Of these, 39,249 were APOE4 carriers, while 99,302 were APOE4 non-carriers.

As shown in Figure 7B, APOE4 carriers exhibited a significantly higher percent of women experiencing early menopause (≤45 years, 17.7%) compared to non-carriers (17.2%, *p* = 0.017). These data in women are consistent with the impact of APOE4 genotype on age of reproductive senescence in the APOE PAM model (Figure 1A). After stratifying the cohort by AD status, women who developed AD had a lower mean age at menopause compared to those without AD (age at menopause in AD: 49.873 ± 5.14 years; age at menopause in non-AD: 50.257 ± 4.81 years; *p*-value = 0.055) (Figure 7C). When stratified by age of menopause, a higher proportion of AD cases were observed in the early (≤ 45 years) menopause groups (20.4% in AD, 15.2% in non-AD, *p*-value < 0.001), whereas menopause >45 years was more prevalent among non-AD women (Figure 7C). Among the confounders of interest, level of education and CVD history resulted significantly different between the AD and the non-AD group (Figure 7C and Supplementary Table 3), therefore were included as covariates in the model. Importantly, while APOE4 carriers exhibited an increased AD risk regardless of menopause age, stratification by menopause age revealed that APOE4 carriers with early menopausal age had the highest AD risk (OR:6.32, 95% CI: 4.78–8.34, *p*-value < 0.001), followed by APOE4 carriers with normal menopause age (OR:4.85, 95% CI: 4.0–5.9, *p*-value < 0.001), followed by APOE4 non-carriers with early menopause age (OR:1.56, 95% CI: 1.1–2.2, *p*-value = 0.008) (Figure 7D), relative to E4 non-carriers with normal menopause age.

Together, these results are consistent with preclinical APOE PAM results, suggesting that the APOE4-age at menopause interaction may contribute to the greater risk of Alzheimer′s in APOE4 postmenopausal females.

## 4 Discussion

Analyses reported herein indicate that APOE genotype impacts midlife endocrine aging. Relative to APOE3, the APOE4 genotype is associated with (1) accelerated endocrine aging and deficits in metabolic reprogramming in brain; (2) greater mitochondrial dysfunction; (3) increased microglia activation; and (4) increased demyelination. In women, APOE4 was associated with early menopause and APOE4 women experiencing early menopause had the greatest risk of Alzheimer′s diagnosis.

The menopausal transition involves complex hormonal fluctuations and multiple system of biology in both the brain and periphery, which traditional ovariectomy (OVX) models do not fully replicate. In contrast, previous research developed a perimenopausal animal model that parallels the human perimenopause to menopausal transition and is mechanistically consistent with female brain imaging outcomes (Yin et al., 2015, Wang et al., 2020b,Mishra et al., 2020, Mosconi et al., 2021, Mosconi et al., 2017a,Mosconi et al., 2018). Interestingly, the APOE3 PAM mouse model outcomes are consistent with the systems biology of endocrine aging identified in the wild-type mouse and rat PAM model (Yin et al., 2015, Wang et al., 2020b,Klosinski et al., 2015, Mishra et al., 2020). In contrast, the APOE4 mouse exhibited accelerated endocrine aging and amplified metabolic dismantling. Relative to APOE3 counterparts, APOE4 females exhibited accumulation of peripheral adipose tissue with high plasma triglycerides and ketone body dysregulation during the perimenopausal transition. Consistent with accelerated dismantling of brain estrogen signaling during the perimenopause, AMPK-PGC-1α-NRF1 signaling declined in APOE4 females, which was accompanied with an inability to mount adaptive metabolic reprogramming in the APOE4 female brain during midlife. Paralleling metabolic shifts, APOE4 females also exhibited greater mitochondrial dysfunction, increased brain demyelination, and microglial activation with a shift toward a pro-inflammatory phenotype. These results provide plausible mechanistic pathways underlying the greater risk of Alzheimer′s in APOE4 postmenopausal females.

### 4.1 Accelerated endocrine aging and disrupted hormone signaling in APOE4 females

Genetic factors play a pivotal role in determining the timing of natural menopause, accounting for 50–60% of the variation in age at menopause (Snieder et al., 1998, Laven, 2015). In our analysis, we found that APOE4 is associated with early menopause in both women and PAM mouse model. This finding is consistent with a previous study reporting younger age of menopause in APOE4 carriers (Koochmeshgi et al., 2004). An earlier age at menopause has been associated with increased risk of age-related disease, including cognitive decline and Alzheimer′s disease (Corbo et al., 2011, Scheyer et al., 2018). Consistently, our analysis confirms that this interaction between APOE4 and menopause may contribute to the greater risk of Alzheimer′s observed in APOE4 postmenopausal females.

Mechanistically, APOE4 9M-Irreg females exhibited higher plasma estradiol and lower progesterone levels compared to APOE3 counterparts. APOE4 is associated with disrupted cholesterol homeostasis, and cholesterol serves as the precursor for steroid hormone production (Martins et al., 2006, Mahley and Rall, 2000). Further, APOE4 PAM mice exhibited increased adipose tissue, which has been shown to influence the balance of plasma estrogen and progesterone levels (Carlson et al., 2012). Therefore, APOE4-dependent dysregulation of lipid metabolism may contribute to the elevated estrogen and decreased progesterone levels observed in APOE4 9M-Irreg animals. This higher-than-normal E2 level in the periphery and increased E2 fluctuation observed here in APOE4 PAM mice is also consistent with the pattern observed in perimenopausal women (Burger et al., 2007, Santoro and Randolph, 2011). Accordingly, APOE4 9M-Irreg and 15M-Irreg females exhibited decreased brain estrogen receptor signaling compared to APOE3 counterparts, potentially due to the inverted U function of E2 dose (Foster, 2012). These results are consistent with a previous report indicating a positive correlation of E2 levels with brain aging in APOE4 carriers (De Lange et al., 2020) and align with multiple lines of evidence indicating that APOE modulates systemic and neural action of estrogen (Valencia-Olvera et al., 2023, Rettberg et al., 2014). Further, both estrogen and progesterone play significant roles in regulating lipid metabolism (Carlson et al., 2012, Mauvais-Jarvis et al., 2013, Rettberg et al., 2014). Thus, APOE4-dependent hormone dysregulation may further exacerbate lipid metabolic disturbances during menopausal transition, contributing to the shift in metabolic phenotype observed in these animals.

### 4.2 Convergence of endocrine aging and APOE genotype on brain mitochondrial function

In APOE3 females, an increase in the expression of genes involved in glycolysis and TCA cycle was observed in the Acyc group, consistent with our earlier findings in rat brain (Yin et al., 2015, Wang et al., 2020b). In contrast, decreased brain estrogen and AMPK-PGC-1α-NRF1 signaling was associated with an inability to mount compensatory adaptive metabolic reprogramming in the APOE4 female brain, which was paralleled by a greater decline in brain mitochondrial function. These findings were consistent with previous reports indicating reduced pAMPK levels in APOE4 brains (Wang C. et al., 2023), and aged APOE4 mice exhibit decreased hippocampal and cortical mitochondrial respiration (Area-Gomez et al., 2020) and reduced brain OXPHOS gene expression (Shang et al., 2020), all of which can contribute to AD pathogenesis (Brinton et al., 2015, Yao et al., 2009). Translationally, these findings in the APOE4 mouse are consistent with disrupted mitochondrial function, detected in postmortem AD brain (Yin et al., 2020). In addition, transcriptomic analysis indicated that both APOE4 9M-Irreg and 15M-Irreg females exhibited elevated expression of H2ac20 (H2A clustered histone 20) compared to their APOE3 counterparts (Supplementary Figure 1). APOE4 9M-Irreg females exhibited reduced expression of *Them5* (Thioesterase superfamily member 5, a mitochondrial protein involved in long-chain fatty acyl-CoA metabolic process) and *Tshb* (Thyroid stimulating hormone subunit beta) compared to APOE3 9M-Irreg females, suggesting dysregulation of lipid metabolism and thyroid hormone regulation (Supplementary Figure 1). Collectively, these data indicate that the female APOE4 brain is compromised in its ability to mount metabolic reprogramming following the loss of estrogen to sustain the metabolic demand of the brain.

### 4.3 Increased neuroinflammation in APOE4 female brains

The menopausal transition is associated with dynamic changes in neuroimmune profile, which can contribute to an increased AD risk in women (Brinton et al., 2015, Mishra et al., 2020, Mishra et al., 2022). Specifically, perimenopause is characterized by increased inflammatory gene expression in the brain (Yin et al., 2015, Mishra et al., 2020). Herein, relative to APOE3 mice, APOE4 mice exhibited an exacerbated and persistent pro-inflammatory phenotype during endocrine aging, which may also be related to the APOE4-driven lipid dysregulation (Parhizkar and Holtzman, 2022, Rebeck, 2017). Specifically, APOE4, PAM females exhibited decreased IL-10 and increased IL-1β levels, microglial activation and upregulation of multiple cytokine pathways, compared to APOE3 counterparts. IL-10 is an anti-inflammatory cytokine and plays a central role in limiting the immune response and promoting survival of brain cells in most major diseases (Strle et al., 2001). In contrast, chronic microglia activation and increased pro-inflammatory IL-1β levels induce neuronal damage and exacerbate Aβ plaque accumulation, thus contributing to AD pathogenesis (Wang et al., 2015, Shaftel et al., 2008). Clinical observations demonstrated that APOE4 carriers exhibited greater microglia activation, lower IL-10 levels, and higher IL-1β levels (Fan et al., 2017, Galgani et al., 2022, Ferrari-Souza et al., 2023). Replicating clinical findings, our results indicated that sustained neuroinflammation initiated by menopause and amplified by APOE4 genotype could contribute to the increased AD risk in APOE4 females.

### 4.4 Alteration in myelination in APOE4 female brains

Our previous findings indicated that white matter catabolism in the perimenopause to menopausal female brain is accompanied by upregulated myelin-related gene expression and elevated myelin and lipid metabolism (Klosinski et al., 2015). Herein, our results demonstrated that APOE3 mice exhibited a transcriptional profile consistent with the wild-type PAM mice. Specifically, upregulation of myelin-related gene expression occurred during the perimenopause, which diminished in the postmenopause but did not revert to premenopausal level.

Compared to APOE3 counterparts, APOE4 PAM females exhibited earlier and persistent upregulation of myelination-related genes, which was accompanied by decreased myelination in the brain. These results align with the association of APOE4 with disrupted cholesterol homeostasis (Martins et al., 2006) and impaired myelination (Blanchard et al., 2022), indicating the synergistic effects of menopause and APOE4 on brain myelination. Upregulation of genes related to myelination is associated with aging-related cognitive impairment (Blalock et al., 2003, Kadish et al., 2009, Rowe et al., 2007). Recent single-cell transcriptomics further indicated increased expression of genes related to myelination in multiple cell types in AD patients (Grubman et al., 2019, Mathys et al., 2019). Myelin dysfunction has been shown to drive amyloid-β deposition in AD mouse models (Depp et al., 2023). The upregulation at the transcriptional level may represent a compensatory response to myelin loss in AD. Together, our results provide insights into the decreased whiter matter integrity observed in aged APOE4 females (Shang et al., 2020).

While Alzheimer′s disease is not unique to the female, findings reported herein provide a mechanistic rationale for the impact of APOE4 during midlife endocrine aging that could increase the risk of AD in later life. Understanding these processes in women could lead to greater understanding of the mechanisms driving the disease, contribute to early identification of those at greatest risk for AD, and lead to interventions to prevent, delay and treat AD.

## Data Availability

The datasets presented in this study can be found in online repositories. The names of the repository/repositories and accession number(s) can be found below: https://www.ncbi.nlm.nih.gov/geo/query/acc.cgi?acc=GSE282066.

## Supplementary material

Supplementary Figure 1 Volcano plots of the top 10 genes exhibiting highest magnitude of log2 fold changes in the APOE4 9M-Irreg **(A)** and 15M-Irreg **(B)** groups compared to their APOE3 counterparts.
