# Supplementary figures and images for: Accelerated midlife endocrine and bioenergetic brain aging in APOE4 females
Source: Front Aging Neurosci. 2025 Aug 18;17:1632877. doi: 10.3389/fnagi.2025.1632877 (PMC12399568; doi:10.3389/fnagi.2025.1632877)

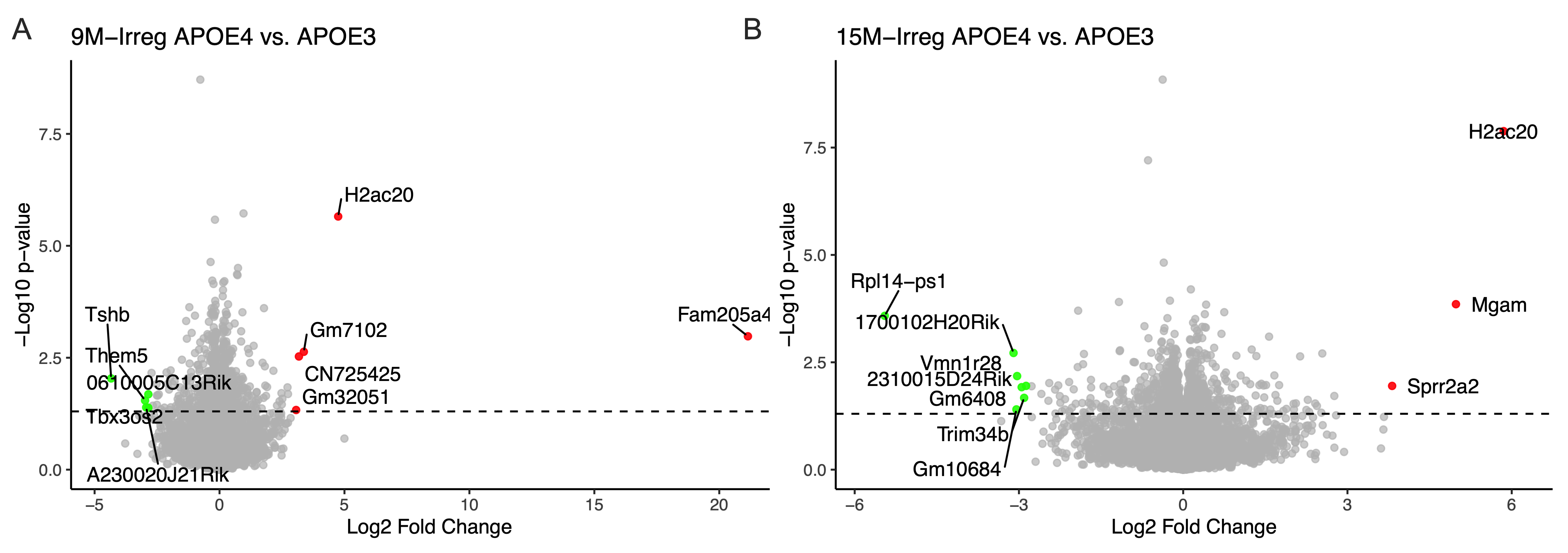

Supplement: Supplementary Figure 1 — Volcano plots of the top 10 genes exhibiting highest magnitude of log2 fold changes in the APOE4 9M-Irreg (A) and 15M-Irreg (B) groups compared to their APOE3 counterparts. [file Image_1.tiff]
