# Supplementary material for: Accelerated midlife endocrine and bioenergetic brain aging in APOE4 females
Source: Front Aging Neurosci. 2025 Aug 18;17:1632877. doi: 10.3389/fnagi.2025.1632877 (PMC12399568; doi:10.3389/fnagi.2025.1632877)
Supplement: Supplementary file 2 [file Table_1.docx]

Supplementary Table S1 P-values for the nested model.

|  | **Fig 1B. E2** | **Fig 1C. P4** | **Fig 1D. Uterine weight** | **Fig 2A Body weight** |
| --- | --- | --- | --- | --- |
| **Genotype** | 0.00719298 | 0.132359619 | 0.199041198 | 0.04153916 |
| **Age** | 0.000622254 | 0.098662324 | 3.7716E-06 | 2.23023E-15 |
| **Cycling_nested** | 0.039154191 | 0.000548667 | 0.551901936 | 0.064888233 |
| **Genotype:Age** | 0.534896942 | 0.183424842 | 0.050316444 | 0.073322205 |
| **Genotype:Cycling_nested** | 0.712140725 | 0.636507048 | 0.121547978 | 0.250345259 |
|  |  |  |  |  |
|  | **Fig 2B Adipose index** | **Fig 2C Muscle mass** | **Fig 2D Glucose** | **Fig 2E Triglyceride** |
| **Genotype** | 0.000310107 | 0.000122725 | 0.737872258 | 0.00254009 |
| **Age** | 2.4511E-08 | 3.51101E-10 | 0.822551195 | 0.034391459 |
| **Cycling_nested** | 0.742451796 | 5.3909E-41 | 7.83528E-07 | 5.83509E-05 |
| **Genotype:Age** | 0.006347636 | 0.000323365 | 0.859015203 | 0.997175232 |
| **Genotype:Cycling_nested** | 0.039199402 | 0.007980585 | 0.439447839 | 0.59537742 |
|  |  |  |  |  |
|  | **Fig 2F Ketone body** | **Fig 3C Ppargc1a** | **Fig 3D Nrf1** | **Fig 4A mtDNA** |
| **Genotype** | 0.392163303 | 0.058878955 | 0.247271972 | 1.431E-10 |
| **Age** | 0.004185139 | 1.43274E-05 | 0.01361428 | 2.50776E-06 |
| **Cycling_nested** | 7.97872E-05 | 1.88258E-17 | 2.24959E-19 | 1.09899E-18 |
| **Genotype:Age** | 0.061461612 | 0.178111543 | 0.011436165 | 0.199891438 |
| **Genotype:Cycling_nested** | 0.059502438 | 0.152181993 | 0.018945186 | 0.949698646 |
|  |  |  |  |  |
|  | **Fig 5A Aif** | **Fig 5D IL-10** | **Fig 5E IL-1b** | **Fig 5F IL-6** |
| **Genotype** | 0.417211328 | 0.011183834 | 0.001485077 | 0.003695999 |
| **Age** | 9.88481E-05 | 0.270392935 | 0.005798279 | 0.004720127 |
| **Cycling_nested** | 0.039596084 | 8.28759E-07 | 0.010511197 | 1.08088E-05 |
| **Genotype:Age** | 0.00745132 | 0.131662416 | 0.012685931 | 0.010238211 |
| **Genotype:Cycling_nested** | 0.060875961 | 0.06006248 | 0.071711581 | 0.033573068 |

Supplementary Table S2. P-values and effect sizes for significant two-way ANOVA results.

|  | **P value** | **Partial Eta-squared** |
| --- | --- | --- |
| **Fig 1B. E2** |  |  |
| **Genotype** | 0.002334195 | 0.144194589 |
| **CEA** | 0.015529977 | 0.182119375 |
| **Interaction** | 0.708223785 | 0.03463554 |
| **Fig 1C. P4** |  |  |
| **Genotype** | 0.050208088 | 0.088243411 |
| **CEA** | 0.477016235 | 0.07832465 |
| **Interaction** | 0.558321348 | 0.06735143 |
| **Fig 1D. Uterine weight** |  |  |
| **Genotype** | 0.084375891 | 0.043164653 |
| **CEA** | 0.007165547 | 0.184181365 |
| **Interaction** | 0.069831918 | 0.118136902 |
| **Fig 2A Body weight** |  |  |
| **Genotype** | 0.016871981 | 0.069297193 |
| **CEA** | 1.74922E-07 | 0.367328331 |
| **Interaction** | 0.166672234 | 0.076784629 |
| **Fig 2B Adipose index** |  |  |
| **Genotype** | 5.23231E-05 | 0.197154192 |
| **CEA** | 0.000144186 | 0.258469462 |
| **Interaction** | 0.014566165 | 0.150661152 |
| **Fig 2C Muscle mass** |  |  |
| **Genotype** | 1.01285E-05 | 0.230124722 |
| **CEA** | 7.23921E-06 | 0.318824078 |
| **Interaction** | 0.00175171 | 0.202803348 |
| **Fig 2E Triglyceride** |  |  |
| **Genotype** | 0.001354973 | 0.168847102 |
| **CEA** | 0.94399767 | 0.013211252 |
| **Interaction** | 0.654638259 | 0.041984765 |
| **Fig 2F Ketone body** |  |  |
| **Genotype** | 0.436852792 | 0.0075752 |
| **CEA** | 0.049341022 | 0.110905857 |
| **Interaction** | 0.057977939 | 0.10659325 |
| **Fig 3C Ppargc1a** |  |  |
| **Genotype** | 0.043573682 | 0.080540219 |
| **CEA** | 0.010420316 | 0.231878105 |
| **Interaction** | 0.106178672 | 0.141559095 |
| **Fig 3D Nrf1** |  |  |
| **Genotype** | 0.129876028 | 0.046193293 |
| **CEA** | 0.001207797 | 0.303192908 |
| **Interaction** | 0.010852439 | 0.230436688 |
| **Fig 3E PGC1a** |  |  |
| **Genotype** | 0.055351551 | 0.254125712 |
| **Menopause** | 0.764831691 | 0.007127482 |
| **Interaction** | 0.328816106 | 0.073373159 |
| **Fig 3F Glycolysis** |  |  |
| **Genotype** | 0.009584313 | 0.303931176 |
| **Menopause** | 0.117336398 | 0.124080298 |
| **Interaction** | 0.035295452 | 0.212811969 |
| **Fig 3G TCA** |  |  |
| **Genotype** | 0.000173039 | 0.532694013 |
| **Menopause** | 0.435866527 | 0.032271803 |
| **Interaction** | 0.027495576 | 0.230812859 |
| **Fig 4A mtDNA** |  |  |
| **Genotype** | 1.31095E-11 | 0.581762267 |
| **CEA** | 0.000129402 | 0.346494515 |
| **Interaction** | 0.720116072 | 0.037885006 |
| **Fig 4H Complex I activity** |  |  |
| **Genotype** | 0.121581148 | 0.127909112 |
| **Menopause** | 0.025715634 | 0.247218881 |
| **Interaction** | 0.012072246 | 0.302016516 |
| **Fig 4I Complex IV activity** |  |  |
| **Genotype** | 0.041960489 | 0.210345491 |
| **Menopause** | 0.427624515 | 0.035302182 |
| **Interaction** | 0.092408599 | 0.149299571 |
| **Fig 4J Complex I western** |  |  |
| **Genotype** | 0.140934756 | 0.148182768 |
| **Menopause** | 0.006227676 | 0.42477677 |
| **Interaction** | 0.321563422 | 0.070136502 |
| **Fig 4J Complex III western** |  |  |
| **Genotype** | 0.096024894 | 0.185305546 |
| **Menopause** | 0.001716513 | 0.516193285 |
| **Interaction** | 0.333446643 | 0.066889523 |
| **Fig 4J Complex IV western** |  |  |
| **Genotype** | 0.139450913 | 0.149207946 |
| **Menopause** | 0.003702472 | 0.463349274 |
| **Interaction** | 0.36290511 | 0.059427157 |
| **Fig 4J Complex V western** |  |  |
| **Genotype** | 0.001248099 | 0.536686443 |
| **Menopause** | 0.001172833 | 0.540592501 |
| **Interaction** | 0.148839975 | 0.142898686 |
| **Fig 5A Aif1** |  |  |
| **Genotype** | 0.155988459 | 0.040645956 |
| **CEA** | 0.145034828 | 0.127767518 |
| **Interaction** | 0.03716303 | 0.184655979 |
| **Fig 5B IBA1** |  |  |
| **Genotype** | 0.002984401 | 0.47866686 |
| **Menopause** | 0.051228301 | 0.245051076 |
| **Interaction** | 0.798178263 | 0.00482828 |
| **Fig 5D IL-10** |  |  |
| **Genotype** | 0.012309023 | 0.104959042 |
| **CEA** | 0.021806236 | 0.179431756 |
| **Interaction** | 0.083099139 | 0.132514628 |
| **Fig 5E IL-1b** |  |  |
| **Genotype** | 0.000474622 | 0.188383644 |
| **CEA** | 0.00216446 | 0.243658713 |
| **Interaction** | 0.034838713 | 0.158581558 |
| **Fig 5F IL-6** |  |  |
| **Genotype** | 0.001421092 | 0.159668481 |
| **CEA** | 0.018779633 | 0.178810524 |
| **Interaction** | 0.038916217 | 0.154859444 |
| **Fig 6C CNPase** |  |  |
| **Genotype** | 0.113384947 | 0.169250059 |
| **Menopause** | 0.007663295 | 0.408739901 |
| **Interaction** | 0.011326568 | 0.377523475 |
| **Fig 6C MBP** |  |  |
| **Genotype** | 0.03498455 | 0.280192317 |
| **Menopause** | 0.001778031 | 0.513878982 |
| **Interaction** | 0.002559432 | 0.489342504 |
| **Fig 6D MBP-Area** |  |  |
| **Genotype** | 0.007465872 | 0.462276155 |
| **Menopause** | 0.00732163 | 0.463901503 |
| **Interaction** | 0.225870943 | 0.119582104 |

Supplementary Table S3. Smoking status, BMI, and Obesity in AD and Non-AD groups.

|  | Non-AD | AD |  |
| --- | --- | --- | --- |
|  | n (%) | n (%) |  |
| # Participants | 102,380 | 582 | p-value |
| Smoking | | | |
| Yes | 4,419 (4.3%) | 28 (4.8%) | 0.492 |
| Occasionally | 1,820 (1.8%) | 6 (1.0%) |  |
| No | 96,096 (93.9%) | 548 (94.2%) |  |
|  | | | |
| BMI-Visit 1 | | | |
| N miss | 333 | | 0.878 |
| Mean (SD) | 26.748 (4.946) | 26.717 (4.978) |  |
| Range | 12.121 - 68.130 | 17.607 - 47.034 |  |
|  | | | |
| Obesity* | | | |
| Yes | 22,749 (22.2%) | 126 (21.6%) | 0.741 |
| * BMI >= 30 kg/m2 |  |  |  |
